# 

**Published:** 1878-12

**Authors:** 


### CONTENTS.

CORRESPONDENCE.

The Power of Aerostatic Changes upon Abnormal Tissue. *By E. C. Chisholm* .......... 483

The Dental Pulps. *By Wm. Pease* .......... 487

Arsenious Acid in Dentistry. *By J. Hardman* .......... 499

An Index Rerum of Dental Literature. *By D. C. Hawxhurst* .......... 509

Materials for Fillings. *By De-Ce-Aich* .......... 512

SELECTIONS.

Hard Rubber Applaince for Congenital Cleft Palate. *By Thomas Brian Gunning* .......... 513

EDITORIAL.

Close of the Volume .......... 522

Lists of Societies .......... 522

Something New .......... 523

Virginia State Dental Society .......... 524

